# The Hospital. Nursing Section

**Published:** 1904-09-17

**Authors:** 


### The Hospital.

### Nursing Section.

Contributions for this Section of "The Hospital" should be addressed to the Editor, "The Hospital," Nursing Section, 28 & 29 Southampton Street, Strand, London, W.C.

No. 938.—Vol. XXXVI. *SATURDAY, SEPTEMBER 17, 1904.*

### Notes on News from the Nursing World.

### OUR CHRISTMAS DISTRIBUTION.

We are anxious to be able, in our special number for October, to state that parcels of articles of clothing for our Christmas distribution have been received; and from that date we hope that our readers will render it necessary for us to announce contributions in every issue until we hold our usual exhibition. Each year the number of patients in hospitals and infirmaries increases, and therefore each year we feel it essential to emphasise our appeal to the generosity of the kindly and compassionate. All past experience teaches us that the more urgently we plead the cause the more generous is the response, and once again we ask that this year there may be a record quantity of parcels. The larger the parcels the better; but a single garment, however small, will be always acceptable. All contributions should be addressed to the Editor, 28 and 29 Southampton Street, Strand, London, W.C., with "Clothing Distribution" written on the outside of the parcel.

### QUEEN ALEXANDRA'S MILITARY NURSING SERVICE.

We are officially informed that Misses G. M. Allen, K. M. Bulman, M. E. M. Grierson, A. M. M. Denny, E. M. Fairchild, E. Foster, O. M. Griffin, E. M. Lyde, A. M. MacCormac, A. M. Orchard, M. S. Ram, and M. F. Steele have been appointed staff nurses in Queen Alexandra's Imperial Military Nursing Service. The first three have been posted to Portsmouth, the second seven to Netley, and the third three to Woolwich.

### THE ROYAL NAVAL NURSING SERVICE.

It will be learnt with interest in the nursing world that Sir Henry Norbury, who has for so many years been Director-General of the Medical Department of the Navy, and in that capacity has been officially responsible for the nursing arrangements of what is now known as Queen Alexandra's Royal Naval Nursing Service, is succeeded by Inspector-General Ellis. The new Director-General entered the Navy as a surgeon nearly 30 years ago, and after serving in several important capacities was advanced to his present rank as Inspector-General of Hospitals and Fleets in February last. As surgeon to a battalion of Royal Marine Artillery he received his first and only campaigning experience during the operations against Arabi Pasha in 1882, on which occasion he was present in the two engagements at Kassassin, on August 28 and September 9, and also at the battle of Tel-el-Kebir, obtaining mention in despatches and promotion to the rank of staff surgeon. He is one of the survivors of the ill-fated *Victoria*, which was sunk off Tripoli in collision with the *Camperdown* on June 22, 1893, when upwards of 370 officers and men perished.

### THE QUESTION OF PRELIMINARY TRAINING.

In an interview with our commissioner, which appears in another page, Miss Helen Todd, matron of the National Sanatorium for Consumption at Bournemouth, maintains that the training which is given at that institution is useful preparation for general training, and confesses that she has often wished that she had received some preliminary experience herself before she entered St. Bartholomew's Hospital, although she might possibly, we think, have found that at St. Bartholomew's previous experience was not considered desirable in a candidate for the post of probationer. We have always appreciated the objection of a matron of a general hospital to engage probationers who because they have been for a year or two in a special hospital have preconceived ideas which it may be as necessary, as it is difficult, to remove. But on the whole preliminary training in a well-conducted special institution should be regarded as an advantage rather than as a drawback. The work which has to be done by the nurses at the National Sanatorium must, at any rate, render it easier for them than for absolutely untrained persons to get through the daily round of duties in a general hospital; and in these days, when open-air treatment is so often a feature at the latter, they ought not to find that their practical knowledge, which cannot be disputed, is looked upon in the light of a disqualification.

### SICK PAY FOR 100 WEEKS.

The value of the sick-pay policies issued by the Royal National Pension Fund for Nurses is illustrated, in a striking manner, by a case which has come under our notice. Nearly two years ago a nurse was knocked down by a cyclist, and in consequence of the severity of her injuries she has since received sick pay continuously for 100 weeks, and is still receiving it. It is obvious that, however confident a nurse may be of enjoying immunity from sickness, the utmost care cannot secure her exemption from the risk of injury due to accidental causes. As this risk is, of course, covered by the sick-pay policy of the fund, we cannot too strongly urge upon nurses who have not yet taken advantage of the benefits offered the importance—in their own interests—of being in a position to claim an allowance which, in the event of an unexpected disaster, will render them independent of extraneous help.

### REGISTRATION IN THE UNITED STATES.

The nurses in Louisiana, who are opposed to State registration and control, have carried the day. In consequence of the presentation of a printed petition signed by 116 nurses expressing strong hostility to the proposed legislation, the Senate Committee on Health and Quarantine declined to proceed with it. As the number of nurses who had advocated the Bill was only 78 and 23 of these were graduates of other States than Louisiana, their decision is not surprising. But if the nurses who did not approve the measure had not exerted themselves and made it clear that their opinion was that of the majority, the Louisiana State Nurses' Association, with only a minority at their back, would have had their way. It is one thing to admit that if an overwhelming majority of nurses demand registration it ought to be conceeded, and quite another to grant it because a small number of active persons professing to represent the profession clamour for it.

### THE VICTORIAN TRAINED NURSES' ASSOCIATION.

At the annual meeting of the Royal Victorian Trained Nurses' Association, Miss Glover in seconding the motion for the adoption of the balance-sheet, felt compelled to call attention to the fact that a good many members had not paid their subscriptions, from which she drew the conclusion that they have ceased to take any interest in it. We agree with her that "indifference is the worst thing that could happen to the association." Miss Glover also deplored the inability of the Victorian nurses to send a direct representative to the recent congress at Berlin, and said, "It is true that Mrs. Bedford Fenwick is asked to represent us, but I think that is rather a feeble way of doing it. We have nobody here holding any position in a Victorian hospital who is able to go to that great gathering of nurses and other women workers, and I regret to say that we have nobody who has sufficient enterprise to start a subscription to enable someone to go." However, Miss Glover consoles herself by the hope that at the next quinquennial meeting, which will probably take place in Canada, the association will be in a position to send its own representative.

### A SUPPOSED NURSE IN THE POLICE COURT.

At the Westminster Police Court on Monday, a woman, dressed as a nurse, who was remanded on a charge of theft, was alleged by a witness to have stated to her that she was "a nurse at the London Hospital." The case being *sub judice*, we, of course, make no comment on it except to say that the statement in reference to the London Hospital is not correct. We understand that no person of the name given by the accused either is or has been, on the staff of that institution.

### NINETEEN WORKHOUSES WITHOUT A NIGHT NURSE.

Mr. Philip H. Bagenal, the Local Government Board Inspector for the East and West Ridings of Yorkshire, in his Nursing Returns for the official year, reports that "in 19 workhouses it has not yet been found possible to provide a night nurse." This shows that, in spite of great improvements in workhouse nursing, much remains to be done, even in Yorkshire, which is supposed to be well abreast with the times. We note with surprise that while at Hull the patients averaged only 8 per nurse, and at Sculcoates 11, the average at York was 19. In the West Riding the average was 12, but at Goole it was 24, at Holbeck 26, and at Selby 35. At Selby there were no pauper attendants. The total number of sick inmates of workhouses, other than imbeciles and epileptics in special wards, in the two Ridings was 4,949; the number of patients to each nurse on the average was 11·69, and the total number of pauper attendants 84.

### THE SLOW DISAPPEARANCE OF THE PAUPER ATTENDANT.

In many respects the nursing return of Mr. James Lowry, the Local Government Board inspector for the counties of Durham, Northumberland, and the North Riding of Yorkshire and four unions in the county of Cumberland is satisfactory. The number of the sick in the district, exclusive of imbeciles and epileptics, was 2,901, as compared with 2,832 in the preceding year. These were attended by 137 day nurses, as against 119 in 1903, while the number of night nurses rose from 34 to 45, giving a total of 182, as against 153. In other words, the average number of patients per nurse was as nearly as possible 16, compared with 18 51. In 1903 the number of pauper attendants was 208, and in the present year it has fallen to 197. This is an improvement, but the return none the less strongly confirms the impression that the entire disappearance of the pauper attendant will not be accomplished for a considerable period, unless the Local Government Board, taking the matter into their own hands, absolutely prohibit the employment in nursing of persons who have no qualifications for the work.

### NURSING ORGANISATIONS AND SUNDAY CONCERTS.

Canon Nunn, of Manchester, has protested against having Sunday concerts in a theatre on behalf of the Ardwick Nurses' Fund. We are reluctant to express disapproval of any particular movement having such an excellent object in view as financial assistance to a nursing organisation; but, on the general question, we think that efforts of this kind, however well meant, should not be encouraged. Apart from the question of Sunday observance, we doubt whether associations which expect to be helped by the clergy and other ministers of religion, have anything to gain in the long run pecuniarily by the opening of places of entertainment at which, if money is not paid for tickets, it is practically given for admission to the performance.

### THE QUEEN'S NURSES AT HUDDERSFIELD.

At an interesting gathering the other day, Mr. Dalton, the retiring honorary secretary of the Huddersfield and District Victoria Sick Poor Nurses' Institution, was presented with a handsome vase as a small mark of appreciation of the work he has done. Some significant figures were cited by the recipient. Mr. Dalton, who has been honorary secretary of the Association since it was founded seven years ago, said that during that period the number of cases attended by the Queen's nurses had been 3,773, the number of operations 600, and the number of visits paid 80,547. The sum raised in subscriptions and donations was £3,800, and while the Association started with only one nurse, there are now a superintendent and five nurses. Mr. Dalton has certainly, by his energy and tact, had a great deal to do with this development, and he has the satisfaction of knowing that he leaves the organisation in a healthy condition.

### SANDALS FOR NURSES.

At the Ararat District Hospital in Victoria the matron has adopted the use of the sandal both for herself and for the staff. She states that it has proved a decided boon for those who suffer from corns, and that the nurses can do vastly more walking, with little or no fatigue. A size smaller than the boots worn is necessary, and the matron recommends the sandals to nurses generally, except in the very wettest weather.

### THE PLEASANTRIES OF DUBLIN GUARDIANS.

It is bad enough when a candidate for a nursing appointment is accepted, or rejected, solely on account of her professions of attachment to a particular religion. But a yet more objectionable test was suggested by one of the guardians of the North Dublin Union who at their recent meeting elected a trained nurse for the Protestant Infirmary. Three candidates attended in person, and the guardian in question proposed that the board should select a lady whose personal appearance best recommended itself to them. The proposal prompted another guardian to ask, "Who was to be the judge of beauty?" These pleasantries may be very amusing to Irish guardians, but they are necessarily offensive to candidates who have to appear before them.

### VILLAGE NURSES' HOME AT SWINDON.

It is proposed to open at Swindon on October 1st a training-home for village nurses. A suitable house has been taken, and a superintendent nurse and a midwife have been appointed by the County Committee. This is the outcome of the public meeting which was held under the auspices of the Wiltshire Nursing Association in April last. The home will, in the first place, be used for training purposes only, but of course the services of the village nurses will be available when the county superintendent considers that, under supervision, they are sufficiently qualified to attend the sick poor.

### RESIGNATIONS AT LEWES WORKHOUSE.

The resignation of another nurse having taken place at Lewes Workhouse the guardians of the Union have agreed to a motion for a return as to the sum they have paid in advertising for nurses, and also for travelling expenses, during the past 12 months. In view of the frequent changes it has also been decided that future applicants shall see the workhouse before they are appointed. If this decision be inflexibly carried out, nurses entering the employment of the Lewes guardians will not, at any rate, be ignorant of the conditions under which they will be required to perform their duties.

### EXETER GUARDIANS AND DISTRICT NURSING.

The Guardians of St. Thomas, Exeter, have lost no time in formulating their scheme for aiding district nursing associations in their district. They have resolved that in making grants the need of the parish and district is to be taken into consideration with special attention to the number and nature of the population and to the difficulties often experienced in scattered rural districts; that attention must be given to the resources of the district and ability to subscribe voluntarily; that no contribution is to be paid unless the nurse is duly qualified to the satisfaction of the district medical officer; that the guardians nominate a representative on the nursing association; and that all applications for subscriptions shall be referred to a permanent committee for report. The permanent committee consists of six members, including a lady.

### EXCESSIVE TRAINING AT A CHILDREN'S HOSPITAL.

It is assumed that in future each probationer of the Children's Hospital at Boston, U.S.A., will give her whole time for four months to studies at her college and instruction in anatomy, physiology, chemistry, bacteriology, and sanitation. Then for two months, at the nurses' home and the hospital, courses will be given in domestic science, cooking, and the essentials of practical work in the hospital. After this six months' period of probation will follow the usual three years' course of hospital training. From the point of view of those who propose to devote themselves exclusively to the nursing of children all this is excellent, but for those who intend to go in for general nursing subsequently there are two serious disadvantages. One is the length of time occupied in the studies and training, and the other that it is not helpful to the general nurse that her attention should be concentrated for three years and a half on a special branch of work.

### NEW REGULATIONS AT ELHAM WORKHOUSE.

Some new regulations in regard to the nursing staff at Elham Workhouse have come into operation this month. The day nurses are now required, at the instance of the Elham Guardians, to assist in cooking if necessary, to also serve meals, and to see that those inmates who are unable to feed themselves are properly fed. Another new regulation provides that the nurses shall "report to the master or matron and obtain a necessary pass on leaving the house"; and leave of absence is now given, except upon extraordinary occasions, once in each week, from 11 a.m. to 9.30 p.m., and every alternate Sunday. When Sunday is impracticable an extra weekday from 11 a.m. to 9.30 p.m. is given.

### A SUCCESSFUL CHURCH PARADE.

The members of the various friendly societies at Buckingham must be congratulated upon the success of their Sunday parade and demonstration. No fewer than 2,000 persons, including the members of the societies with their bands, who had marched through the principal streets of the town, assembled in the Market Square for open-air service, which included an address by the vicar and a short speech by the mayor. The collectors, who had previously obtained considerable sums as the procession moved along its course, then made a final effort, and when the boxes were opened at the Oddfellows' Hall, it was found that nearly £16 had been obtained. Although the Nonconformist ministers did not attend the service, representatives of every religious body were present on the occasion.

### The Nursing Outlook.

"From magnanimity, all fear above;
From nobler recompense, above applause,
Which owes to man's short outlook all its charm."

### QUALITY OF TRAINING.

As a rule the more a nurse pays for her training the worse it is, and at this time of the year, when so many inexperienced young women are seeking to become nurses, it is worth while to warn them against the so-called training in cottage hospitals where premiums are required.

To begin with, most cottage hospitals have only from 10 to 30 beds, and few of these occupied; and as there is no operating theatre the best surgical cases are sent to the nearest big town, and the severe medical cases as a rule go also. So that a probationer might spend many years in a small cottage hospital without ever seeing a major operation or a case of tetanus or typhoid.

Yet, strangely enough, the "training" in a cottage hospital is always briefer than elsewhere. Here is the Boscombe Hospital advertising for a probationer for one or two years and "certificate given," and the Wisbech Hospital also for two years and "certificate given," but no salary; or here is the Paulton Memorial Hospital offering "good surgical training, one year, premium £20." According to "Burdett's Hospital Annual," there are 11 beds at Paulton.

Also these small hospitals, in their effort to get cheap help, reduce the age limit. The Ilfracombe Hospital and the Pentre Fever Hospital advertise for probationers not under 20, and at least a dozen others do the same. Then small special hospitals advertise that they give training, and do not state that they are only "a home for the dying" or a consumptive sanatorium. But the private "homes" are the worst of all; one advertises for a probationer for two years' training, no salary, but "board and lodging provided during second year." Evidently for the first year this "lady" has to live out and yet give her daily quota of work without pay. And of course the theoretical teaching, the lectures and classes, cannot be worth much in most of these small places, even if they are attempted at all. In a cottage hospital of 20 beds where there is only a matron and a probationer and one has always to be on duty, how are they both to sit down, the one to teach and the other to learn? And where are the lectures by eminent medical and surgical specialists such as can be had in the big London and Edinburgh and Dublin schools?

We have every sympathy with the small struggling hospitals which find it difficult to pay their way, but at the same time we have the honour and efficiency of the nursing profession very much at heart, and we say deliberately that it is little better than a fraud for small cottage hospitals to ask young women to pay to be "trained" as nurses within their one or two little wards, for as a matter of fact an efficient all-round nurse cannot be developed without wider experience. Of course the lowering of age, and the shortening of the period of training are all done to tempt the ignorant girl. But it is a pity to do things like this in the name of charity, and all governors and subscribers of small hospitals should stand out against it. It is fair neither to the patients nor the so-called probationers; and the feeling aroused by such a system must lower the whole tone of the hospital and those connected with it. Remember that at the big nurse-training schools no premium is asked; the training is for three years; salary and uniform, and board, lodging, and washing are supplied through all the training; and there are weekly lectures and classes as well as wide experience. Very often the governors and hon. secretaries of the small hospitals do not understand what training of nurses means, and very often they never see the advertisements that are sent out. But the matron and the medical officers must know, and it is their duty to put the matter plainly before their board and ask them if they consider it honest to take a premium from some girl for such experience as she can get in their tiny institution. For it is only "experience," and that of a very limited nature. If your rich damsel thoroughly understands this, and is willing to pay for light work; and if the medical officers and patients are content with such nursing, then it is all right. But it is not "training."

Of course these hospitals ought to be nursed entirely by trained women for the responsibility is so much greater where there is no resident medical officer and no sisters. And the rather larger hospitals of 30 to 60 beds, and the special hospitals ought to combine together to offer a three years' training with one year in each; for instance, a little surgical hospital near mines or works and 100 in-patients which can show 200 casualty cases a year might combine with a cottage hospital and a special London hospital: arrangements being made that whilst at the special hospital lectures on general nursing and preparation for examination should also be included. Except under some such schemes real training in small hospitals there is none, and even this scheme might not be found to work.

Therefore it is well to warn all would-be nurses, that if they wish to succeed in their profession and become eligible for well-paid posts, they must train at a proper "nurse-training school" connected with a hospital of over 100 beds, and where lectures and classes are held, they must not be misled by short cuts to certificates. It is true that it is difficult to get into the best hospital schools, but it is not so difficult to get into the infirmaries and some of these —such as Marylebone and Birmingham—now offer excellent experience and recognised certificates.

### Lectures upon the Nursing of Infectious Diseases.

By F. J. Woollacott, M.A., M.D., B.S.Oxon, D.P.H., Senior Assistant Medical Officer, Park Hospital, Metropolitan Asylums Board, Hither Green.

### LECTURE V.—THE COMPLICATIONS OF SCARLET FEVER.

In this lecture we shall consider those complications of scarlet fever which are the result of extension of the inflammation from the throat to the surrounding structures, and it is well to have a clear idea first of all as to the connection and relationship of these parts the one with the other. On looking into the mouth we see at the back the two tonsils, and between them the soft palate and uvula, hanging like a curtain. Farther back is a space known as the pharynx, into which there are several openings. In front, just above the level of the mouth, are the two posterior openings of the nose, below are the gullet and windpipe, and on each side are the two small Eustachian tubes leading to the ears. When, as in scarlet fever, the throat is inflamed, the tonsils and soft palate suffer first, then the inflammation creeps into the pharynx, and, unless it be checked, it may then extend further, forwards into the nose, downwards to the larynx and windpipe, or laterally into the ears. In this way are produced discharges from the nose and ear, and, less frequently, croup from implication of the larynx. The inflammation may also spread by means of the lymphatics to the glands in the neck, and perhaps give rise to an abscess.

We shall take each of these complications in turn and consider the appropriate treatment. The inflammation of the throat itself, though not strictly a complication, for it is an essential part of the disease, will most conveniently come first. The symptom most troublesome to the patient is usually pain and difficulty in swallowing. In younger children these may be so great that food is obstinately refused, in spite of all coaxing. The remedy is to give nourishment by a tube passed through the nose and into the stomach. If the nose be blocked and cannot be cleared, rectal enemata must be tried. Adults and older children should be encouraged to swallow large mouthfuls in gulps. The pain is very little, if at all, greater than with sips, while a much larger amount of nourishment is taken. Our next step must be to subdue the inflammation and to soothe the inflamed parts, and this is best attained by syringing or irrigating the throat with a warm antiseptic lotion. We have a three-fold object in view—to hinder the growth of bacteria, to soften and remove the sticky mucus, and to wash away any exudation that may have formed, or any particles of food that may remain. In syringing the throat the procedure should be as follows:—Two ball syringes with bone or vulcanite nozzles are necessary. They should be squeezed empty and placed nozzle downward in a basin containing the lotion, with which they will fill as they expand. The patient, if a young child, is to be wrapped round firmly with a draw sheet, in order that struggling may be prevented. He should be held either on the nurse's lap or at the edge of the bed, resting on his side with the face turned downwards over a large basin so placed as to receive the lotion as it runs from his mouth. The nurse then takes up one of the syringes and introduces the nozzle between the teeth and injects the lotion on to the swollen fauces, pausing from time to time to allow the child to take breath. The fluid should run out freely and not be swallowed. If the teeth are clenched do not make any attempt to part them. Pass the nozzle back inside the cheek till its point is just beyond the last molar tooth and then syringe. Do this first on one side and then on the other. As one syringe is emptied it should be replaced, still squeezed flat, nozzle downwards in the lotion and allowed to refill, while the other is brought into use. This should be done alternately with each syringe so that there may be no delay. Older children and adults may be treated while sitting up in bed with the head held forwards and downwards over a basin.

Another method of flushing the fauces is by the use of an irrigator. This consists of a vessel containing the lotion, which, when in use is raised a few feet above the level of the patient. It has an opening at the bottom to which a long rubber tube is attached. At the lower part of the tube a spring clip is fixed, and to the end a nozzle is fitted. The latter is inserted into the mouth in the way just described, and when the pressure of the clip is removed the lotion flows on to the fauces in a steady stream. It is allowed to flow for a few seconds, then stopped, then allowed to flow again and so on. This irrigation method is very satisfactory if properly carried out. It is extremely soothing to the inflamed throat and there is no danger of using too much force. Its chief drawbacks are that it does not cleanse the fauces quite as well as syringing, and there is more danger that the fluid will be swallowed. It is better adapted for adults than young children. Whatever method be adopted certain precautions are necessary. Gentleness is essential, for a forcible insertion of the nozzle entails the risk of loosening the teeth or lacerating the gums. If it be passed too far in, it may bruise or seriously damage the swollen fauces. The breathing of the patient must be carefully watched, and at the first sign of choking the operation must be stopped till all is well again. The lotion must not be swallowed. The best way to avoid this is to keep the face well downwards so that the fluid may run out freely. If, in spite of every care swallowing cannot be prevented, the matter should be reported to the doctor, for some of the constituents of the lotion may be harmful.

Occasionally it is necessary to cleanse the throat by swabbing. To do this satisfactorily the patient must open his mouth well and the nurse must have a good light to see what she is doing. The swab should be of cotton wool, soaked in the lotion, and it should be held in a holder. A Spencer Wells pressure forceps will serve admirably. The tongue should be kept out of the way by means of a spatula or some form of tongue depressor. The little operation should be done with extreme gentleness, otherwise damage may result. If performed efficiently much benefit will result from the removal of the thick, stringy mucus.

In the course of a severe attack of scarlet fever it often happens that the mouth and tongue become sore and ulcerated, very much to the patient's discomfort. The remedy is to keep the mouth as clean as possible, to clean the teeth with pieces of soft rag or lint, and to paint the ulcers frequently with glycerine of borax. Troublesome sores and cracks may form on the lips and the adjacent skin. These are best treated with cold cream or zinc ointment, and it is advisable to open the mouth as little as possible during syringing, or the cracks will not heal well.

In the treatment of nasal discharge we rely to a large extent on irrigation or very gentle syringing. The nozzle should just enter the nostril and not be passed further in. The same precautions are necessary as in the case of the throat, and the fluid should run out freely either by the nostrils or from the mouth. It must not be swallowed.

The discharge from the nose is often very irritating, so that it is a wise precaution to smear cold cream or zinc ointment on the nostrils and the upper lip. In this way

### LECTURES UPON THE NURSING OF INFECTIOUS DISEASES—*Continued.*

a protective coating is formed, and sores are prevented. Every care should be taken to prevent the patient "picking" any sores that may be present on the nose or mouth. The hands should be wrapped in lint, and, if other measures fail, splints made of cardboard or similar material should be bandged to the arms, so that the elbow cannot be bent, and consequently the hand cannot reach the face. If "picking" be unchecked troublesome whitlows may form on the fingers, and they, in their turn, may carry the pus infection to other parts.

A very useful method of dealing with the nasal discharge, especially in the later stages, is to plug the nostrils with cotton-wool. Each nostril should be treated alternately, for about 12 hours at a time. Some children do not object to the presence of the plug; others seize every opportunity of removing it, so that some patience and perseverance will be necessary.

We have seen that the inflammation may extend from the throat to the ear. This occasionally results from too forcible syringing of the fauces, and especially the nose, so that the foul discharges are driven up into the Eustachian tubes. As a rule the sequence of events is as follows:—The middle ear first becomes inflamed and pus collects, which, owing to the tympanic membrane, cannot make its way out. The result is pain and rise of temperature. The child will be fretful and, if too young for speech, will often rub its head on the pillow, or put up its hand to the ear. From these signs the seat of the pain may be guessed. Great relief follows the application of a large fomentation to the side of the head. In a day or two, or even less, the pus will burst through the membrane and appear as ear discharge, with speedy cessation of pain. The treatment is by syringing or irrigating. The side of the head should be directed downwards, and the auricle drawn up and back, in order to stretch out the passage. The stream of lotion should be directed forwards and inwards, *i.e.* in the direction of the canal and along its upper wall. The nozzle must not touch the ear, otherwise damage might result, or the return of the lotion be prevented. After syringing the orifice should be lightly plugged with cotton-wool. Do not forget that the lotion may pass through the ear to the throat. If this occur take care that it is not swallowed. The nozzles, after use, either for the nose, ear, or throat, should be sterilised by boiling.

In some cases the inflammation spreads to the surrounding bone. It not infrequently involves the bony prominence behind the ear, called the mastoid process. This becomes swollen, so that the external ear is pushed forward and rendered prominent. The facial nerve, which lies in the bone, may be damaged, causing paralysis of one half of the face. In a few cases the inflammation extends more deeply still and invades the cavity of the skull, giving rise to an abscess of the brain; or the blood in the adjacent veins may clot and suppurate and general pyæmia result. All these conditions are dealt with by operation.

If the glands of the neck are enlarged and painful fomentations or poultices must be applied. If pus forms an incision will be made. Occasionally the whole of the neck becomes swollen, hard, and tender. In such cases extensive sloughing usually results and the life of the patient is in grave danger.

As will be gathered from the above descriptions the nursing of a really bad case is no light matter. The throat, ears, and nose may all need syringing, while sores on the mouth and face must be dressed and fomentations applied to the neck. All this may have to be repeated several times a day. Great care must be taken not to exhaust the patient too much, and he should be soothed and coaxed as much as possible. A skilful and kind nurse will soon win his confidence; he will cease to struggle and will in time learn to appreciate what is done for him. With good nursing he will often get well in comparative comfort; without it he will die, or, if very tenacious of life, may worry through in misery to a long-deferred and unsatisfactory convalescence.

### The National Sanatorium for Consumption.

### INTERVIEW WITH THE MATRON. BY OUR COMMISSIONER.

Like other well-known charities, the National Sanatorium for Consumption and Diseases of the Chest at Bournemouth cannot afford to stand still, and on the occasion of my visit to this delightfully-situated institution a few days ago the workmen were busily engaged on some very important additions which are being made to the building. The extension, which will be completed early next month, not only means, as Miss Helen Todd, the matron, explained to me, ten more beds for patients, but will also greatly improve the accommodation for the nursing staff.

#### The Nurses' Quarters.

"For instance," she said, "instead of the probationers having, as at present, a bedroom divided between two, they will in future have, as all nurses should, a room each."

"They have already, I see, a very comfortable and pleasant sitting-room."

"Yes, but this has been only as the result of the extension. Until very recently they had a wretched room, which is now to be converted into three cubicles for bath-rooms. Their mesent sitting-room was the doctor's, who will have his quarters in the new end of the building. Mine will be proved there also. We shall get six more bath-rooms for patients in consequence of the extension, which likewise includes a new office for the secretary, which was badly wanted. The laboratory, too, will be remodelled, and there will also be a steam disinfector for bedding, etc.

"Does that exhaust the list of structural changes?"

"The front door will now open flush with the long corridor, which runs from east to west of the building, this, we think, being a considerable improvement."

"You have the male patients on the ground floor, and the females on the first floor?"

"That is the arrangement, and the doctor's quarters have always been on one floor, and the matron's on the other. At present the number of patients is 74, and the wards, which are all on the south side, hold from one to four beds."

#### More Nurses Wanted.

"How many nurses do you reckon ought to be in attendance on the patients?"

"There should, in my opinion, be one for every 10 patients. But hitherto we have not had accommodation for the necessary number."

"How many nurses are there, then?"

"Six. When I came here six years ago there was no night nurse, but after a good deal of agitation one was appointed about a year ago, and maintained ever since. When calculating the staff required for such an institution as this it is necessary to remember that one has not to

### THE NATIONAL HOSPITAL FOR CONSUMPTION—*Continued.*

allow for nurses' holidays, as the institution closes each year for part of July and August."

"They have their holiday during the time the place is closed?"

"Yes, each nurse has five weeks. With regard to off-duty, they have one long day each month, and if they live near enough they can sleep at home the previous night. They also have from 2 to 4.30, or from 5 to 7.15 every day; on Sunday from 2 to 5, or from 5 to 9.30; and every sixth Sunday they go off duty at 2 P.M. and do not come on again that evening."

### Hours on Duty.

"When are they required to enter the wards?"

"The probationers, of whom there are four, take the temperature of the patients between 7.30 and 8 A.M. A cup of tea and bread and butter are taken to each of them by one of the maids who calls them. I think it desirable. At 8 they all have breakfast in the dining-room, the sister presiding, and at 8.30 they give the patients their breakfast. They are not off duty until 9.30 P.M., when the wards are closed, but if they have long hours, they have also a good spell off duty. A full hour is allowed for dinner, and half an hour for tea."

"You speak of probationers. What is the length of the probationary period?"

"It is practically a year. The probationers come here when they are about 19 or 20, and, if they choose and are suitable, they can stay on until they are old enough to enter one of the big London or provincial hospitals. As a rule, they do very well afterwards."

"How about teaching?"

"I give them lectures on elementary physiology and anatomy, and on nursing, alternately; and I make a point of teaching them to do what they can in the way of cooking. With a small staff under my own eye it is possible to do this. A very important part of their duties is to see that things are kept clean; and there is, therefore, a good deal of sweeping, polishing, and dusting. On the other hand, as we have quite an army of maids—10, as well as a porter and a boy in the winter—when we have all coal fires—they do not have much rough work."

"The sister, of course, is a trained nurse?"

"Both the sister and the charge-nurse. I have a great horror of swelling the ranks of untrained nurses; and though when our probationers leave I give them a testimonial, they receive nothing in the shape of a certificate. This point is carefully explained to every probationer before her engagement."

### Breeding-Ground for Untrained Nurses.

"I am sure," continued Miss Todd, "that these small institutions are a great breeding-ground for the untrained nurse, unless the utmost vigilance is exercised, and the fact that they are so numerous is one of the reasons why I am in favour of State registration. Under the existing condition of affairs, girls enter such institutes for a short period—in some cases not even for a year—receive a certificate, and forthwith consider themselves nurses. Many of them, armed with their so-called certificate, are employed by nursing institutions, and do a vast amount of mischief to nursing as a profession. This is not the worst, for there are private nursing homes which take premiums from girls who are supposed to be probationers. What can a girl learn in a private nursing home as a probationer? She does not always get much practice, and as to theory, it is not taught. Registration would, I believe, be a remedy for these evils."

### Compulsory Registration.

"I gather, then, that you would not be satisfied with permissive registration?"

"No, it would be futile if it were not compulsory. Of course, it would be fair to place nurses who have been working four or five years on the register, but I would not have any new nurses registered unless they were fully-trained and possessed a certificate to that effect. As a council of perfection, I favour a State examination, as well as the appointment of a general nursing council. We cannot expect to obtain everything at once, but I am convinced that something ought to be done in the direction of protecting fully-trained nurses from the evil effects caused by the multiplication of half-trained persons, and the public from being misled by them."

### Diet and Stimulants.

"Reverting to the teaching of cookery, diet is here, of course, a matter of the most careful study?"

"The treatment is very much a question of diet, and the matron who, in an institution of this kind is necessarily a sort of general factotum, has to give it her constant and most anxious attention. It is one of the essential obligations of the nurses to see that the meals of the patients are nicely served. The food is not weighed, but nurses are expected to induce the patients to eat as much as possible. The patients see the joints, and have a choice of two. There are also two puddings to choose from. The object is, in short, to tempt the palate, and nurses can do a good deal in this way. Stimulants are not given except when specially ordered, but the patients may drink as much milk as they like."

### The Question of Infection.

"Do you care to make any remarks on the subject of infection?"

"I may say that there is not the slightest fear of any one being infected owing to the existence of the National Sanatorium. Some people go to the most absurd extremes in their apprehension, lest they should expose themselves to the risk of contracting phthisis. I have had persons ask to see me at the garden gate because they were afraid that if they came inside the grounds they might be infected. As to the staff, neither a single nurse, nor a single servant, has ever taken the disease here. Some of the servants have been in the Sanatorium for several years; the cook for nine."

### The Open-Air Treatment.

"You include the days before the open-air treatment was introduced?"

"I do. Even prior to the time we adopted that treatment, there had not been a case. The open-air treatment, which was introduced after I came has, however, revolutionised the arrangements. All the windows in exposed positions were double and only allowed a very limited opening, most of them had curtains before them. Now, as you have seen, the casements open in five sections, the top one being a fanlight; this arrangement permits the opening of the greater part of the window even in the most inclement weather. This system prevails throughout the building."

### Ventilation and Rest Rules.

"I suppose the ventilation has also been made more thorough?"

"Lately, we have knocked holes through the main walls into the corridor. All the rooms are also ventilated through the chimney; and the open fireplaces are an effective means of ventilation. As a small detail I may point out that the doors have a hook and eye arrangement, so that they can be fastened back and so avoid banging."

"Do the patients spend much time in the grounds?"

"During the day they are either in the shelter or on the lawns, the sexes being always separated, except when they meet at the services in the chapel. In addition to holding various services in the chapel, which is connected by a cloister with the Sanatorium, the chaplain comes twice a day into the wards to read prayers."

"The nurses, I presume, have to see that the patients take their prescribed rest?"

"Yes, that is an important part of the treatment. The patients are obliged to be on the couches or long chairs an hour before and an hour after the principal meals. Recently we have made it a rule that every new patient should deposit a shilling and have a thermometer for his or her own use. If the thermometer is in a good condition when they leave the shilling is returned."

### Special Regulations.

"What special regulations do the nurses have to see enforced?"

"A pocket-spittoon flask is used in the daytime and ordinary hospital mugs at night. Patients are not allowed at night to put their handkerchief, which is of Japanese paper, under the bed-clothes. Little bowls standing on each locker are provided for that purpose. The flasks and mugs are all boiled once a week by one of the nurses."

"Do you find it essential to select strong young women as nurses?"

"No, I have had two old patients as nurses. They have both done exceedingly well, and are now in excellent health."

"Is outdoor uniform worn?"

"It is optional. With regard to the work, I should say that it is not hard, and that as a rule the nurses are not more than pleasantly occupied. Now and again there is a rush of work owing to acute cases, and when this occurs I am empowered to obtain extra nurses. With the view of giving my own staff the advantage of all the experience possible, I put them on to the acute cases, and employ the extra nurses to do the regular work."

### The Value of the Experience.

"Supposing that a nurse is anxious to acquire knowledge, she may, I gather, learn a good deal that will help her in the future?"

"Undoubtedly, they may learn enough to help them materially when they go in for general nursing. We do not teach dispensing, but a fair knowledge of the use of common drugs can be obtained. Practically we have no surgical cases."

"You think that the training here is useful preparation for general training?"

"I am satisfied that it is. I have often wished that I had received some preliminary experience before I went to St. Bartholomew's Hospital."

"After your training did you go to a general hospital?"

"No; I worked as midwifery pupil and as locum for the matron and different midwifery superintendents at the Brighton Hospital for Women. After that I was assistant matron for nine months at the Hackney Union School at Brentwood, in Essex. I had entire charge of the children's infirmary, and liked the work immensely. Then I came to Bournemouth."

### The Latest New Departure.

"How does the treatment affect the patients?"

"They nearly all improve, and I should say that the majority of the early cases would recover if they could stay long enough. We have about half-a-dozen free beds, and the patients who come in with a governor's letter, pay 7s. 6d. a week. Further support of the institution is badly needed. Financially, we suffer locally because, owing to the number of patients who come from a distance, we receive only a small contribution from the local Sunday Fund which is distributed on a capitation system. We consider that we have a right to ask for both local and national assistance. Our latest new departure has been the foundation of a Ladies' Household Linen Association, of which I am honorary treasurer. It was very badly wanted, and I am glad to add that the movement has been taken up with enthusiasm. The Association, in three months, has supplied at least £50 worth of household linen and blankets to the Sanatorium."

### Presentations.

Port Augusta Hospital, South Australia.—Miss M. Rogers, on resigning her appointment as charge nurse in the Port Augusta Hospital, has been presented with a hypodermic case and fittings by the nursing staff, with two serviette rings by the maids, and with a pair of sleeve-links by the wardman as tokens of esteem. Nurse Rogers, who has sailed for Western Australia, takes with her the good wishes of all her late colleagues.

### Novelties for Nurses.

By Our Shopping Correspondent.

### PEGAMOID WATERPROOF BED-SHEETING AND JACONET.

Pegamoid waterproof bed-sheeting and jaconet, besides having a nice appearance and supple finish, is remarkably odourless. I have proved that it can be boiled for an hour without being affected in the slightest degree, and also that it successfully withstands the action of carbolic acid, ammonia, solution of perchloride of mercury, and formaldehyde. Nitric acid affects it slightly. Mackintoshes are always an item of considerable importance and expense in every hospital, and Pegamoid waterproof sheeting should be valuable for institution work as well as for domestic use. It is made in four different qualities, and is quite reasonable in price. The manufacturers are "The New Pegamoid, Ltd.," 144 Queen Victoria Street, London, E.C.

### PERI-LUSTA.

Peri-Lusta is a strong and inexpensive substitute for silk in knitting, embroidery, crochet and fancy work generally. It is of great brilliancy and such a good substitute that it practically defies detection. The colours are good, it is generally so satisfactory that I feel that its popularity is assured. Any fancy work repository will supply it if ordered.

### POSTCARDS.

From Messrs. Jarrold and Sons, Limited, I have received some particularly good coloured pictorial postcards, reproduced from original water-colour sketches of London, and including views of the Tower Bridge, the Houses of Parliament, Trafalgar Square, and other places of interest. They can be obtained at most stationers.

### Everybody's Opinion.

[Correspondence on all subjects is invited, but we cannot in any way be responsible for the opinions expressed by our correspondents. No communication can be entertained if the name and address of the correspondent are not given as a guarantee of good faith, but not necessarily for publication. All correspondents should write on one side of the paper only.]

### THE PRIVATE NURSE AND HER DUTIES.

Maude E. Besley, writes:—As a private nurse, and I think I am justified in saying a successful one, I really must protest against the article entitled "A Private Nurse and Her Duties," by a physician. I was much surprised at his statement "as regards the patient." To quote from his letter: "His position in life will probably be quite different from that of the ordinary hospital patient. The nurse must not forget this. At the very outset she must show him due respect." Surely there is more than a suspicion of snobbery about all this. Are not all human beings, when ill and suffering, entitled to our respect and consideration, quite regardless of position? Would any nurse worthy of the name treat a patient less kindly because he happened to be poor instead of rich? No, indeed; hospital patients rather claim more of our sympathy and forbearance, seeing that they are isolated from their friends and relatives. Your correspondent also speaks of the error of attempting to "snub" a private patient. That is a word I dislike. An attempt to "snub" any fellow creature, whether ill or well, rich or poor, is one of the worst signs of ill-breeding. Again, in speaking of the patient's relations he says, "They will constantly come into the room when the patient has just fallen asleep, and wake him up." My experience is that they do nothing of the sort. No nurse in her senses would permit such a thing, neither would the relatives think any better of her if she did. "A physician" doubtless means well, but perhaps I may be pardoned for saying that the majority of even the best "physicians" would make very bad nurses. No, let us each, doctors and nurses, be content to shine in our respective spheres, and the literature of doctors relating to private nursing cannot, in my opinion, be too "scanty."

[This letter was omitted last week by a mistake of the printer.—Ed. The Hospital.]

### A DELICATE QUESTION.

"Observer" writes: I was very interested in the letter of your correspondent, "A Superintendent," concerning the behaviour of private nurses out of doors. I fully sympathise with her, and can assure her that she is not the only superintendent confronted by that difficulty. It is one, however, that I am afraid will be impossible to overcome by any "rules" or efforts of a single individual. The evil is a deeply-rooted one, and the remedy lies in the hands of hospital matrons. In fever hospitals chiefly, but also in others, women are received as probationers who are entirely unsuited to the profession of nursing. Frivolous, ill-educated, and utterly without refinement, they obtain their certificates in two or three years' time, and are foisted on the public as private nurses. Then, escaping from the restraints of hospital life, they proceed to bring discredit upon the uniform they wear and the nursing home they belong to. Until hospital matrons insist upon a higher standard of education and refinement in the probationers they engage, superintendents of nursing homes, and, in fact, the public generally, will have to suffer under what I think I may term a growing evil.

### APPEAL BY THE MATRON OF A LEPER HOSPITAL.

Miss A. M. Whiteman, matron of the Pretoria Leper Asylum, writes: In reference to your "Note" in The Hospital of August 17th, may I mention that I am leaving for South Africa on the 24th of this month instead of by a later boat? Any contributions should therefore be sent in to me not later than the 20th instant, care of Messrs. Bullard, King and Company, East India Docks, London, E.

### Appointments.

[No charge is made for announcements under this head, and we are always glad to receive, and publish, appointments. The information, to insure accuracy, should be sent from the nurses themselves, and we cannot undertake to correct official announcements which may happen to be inaccurate. It is essential that in all cases the school of training should be given.]

Alton Workhouse Infirmary.—Miss Martha Louisa Taylor has been appointed superintendent nurse. She was trained at Bristol Workhouse Infirmary, where she has since been charge nurse.

Chalmers Hospital, Edinburgh.—Miss Lily Emmerson has been appointed sister. She was trained at the Royal Halifax Infirmary, and has since held the post of staff nurse at a surgical home in Edinburgh.

General Hospital, Birmingham. — Miss Alice A. Warriner has been appointed sister. She was trained at the Grimsby and District Hospital and at the London Temperance Hospital. She has been charge nurse at the Croydon Borough Hospital, sister at the Dover General Hospital, sister at the Wolverhampton General Hospital, and holiday sister at the General Hospital, Birmingham.

Lancaster Corporation Sanatorium. — Miss Jessie Allan has been appointed sister. She was trained at University College Hospital, London, and has since done private nursing in connection with the Royal British Nurses Association.

Richmond (Surrey) Union Infirmary.—Miss Lavinia Wilson has been appointed head night nurse. She was trained at the Rotunda Hospital, Dublin, for midwifery. She has since been nurse at the Accident Hospital Hednesford; Cottage Hospital Whitchurch; Medway Union Workhouse; and district midwife at Cambridge.

Royston Hospital, Herts.—Miss Faith Wilson has been appointed staff nurse. She was trained at the Royal Infirmary, Bradford, and was afterwards for three years on the private staff at the Manningham Lane Institution.

### "The Hospital" Convalescent Fund.

The Hon. Secretary begs to acknowledge with thanks the receipt of 5s. per the Travel Correspondent of The Hospital.

### TRAVEL NOTES AND QUERIES.

By our Travel Correspondent.

Season at Salsomaggiore (A. E.).—Your letter came much too late for the last issue. It is necessary to send a query at least ten days before the answer will appear. The season is a long one, from May to November. I do not think it would be a suitable place for articular rheumatism, but such a question can only be decided by the medical man in attendance. It is a most dangerous thing for non-professionals to offer advice in such cases. The other questions you ask are well within my province, so I am pleased to help. The best route is via Dover and Calais (for comfort), but the cheapest is by Newhaven-Dieppe. Bale and Milan approximate cost: First single, £6; second single, £4; first return, £10; second return, £7. The place is situated about 20 miles from Parma. Several new hotels have sprung up, and a visitor must be guided by the depth of his purse, none of the hotels is very cheap. It is difficult to estimate the cost of the treatment, but it probably lasts five or six weeks. At the most moderate computation accommodation for six weeks would come to £15. Then there are the baths, fees at the springs, and doctors' charges, after which there would probably be an after cure in the mountains. Should the doctor advise your friend to go there, and you will tell me how much he can afford to spend, I will try to advise as to the most economical plan.

### Echoes from the Outside World.

### Movements of Royalty.

The King left Rufford Abbey on Monday for Balmoral, and in the course of Saturday he planted a tree to commemorate his visit. When his Majesty arrived at Ballater the flags were flying, and a guard of honour was posted opposite the station. He was met by the Prince of Wales and Prince Edward, and the Royal party at once entered an open carriage and drove off to Balmoral amidst loud cheers from the onlookers. Large numbers of the Balmoral tenantry and retainers were present at the Castle to welcome the King. The Queen, who was delayed in landing at Aalesund on account of the inclemency of the weather, made a donation of £100 to the new Children's Home. She arrived at Geiranger on Sunday evening, and on Monday morning she made excursions in the neighbourhood.

### The War in the Far East.

According to a telegram received from Marshal Oyama in Tokio on Friday, the central column of General Kuroki's army encountered on September 4th a superior Russian force near To-lien-kau, drove them back after a severe fight, and occupied the position. The left column of the same army on September 5th occupied Lan-ni-pu, and the right column occupied the whole of the Yu-men-tse-shan on the same day. The Russian main body have retreated north of the Hun River, and have left over 3,000 corpses in the vicinity of Liau-yang. They succeeded in saving their guns, but blew up a number of waggons containing ammunition. They left, however, a large quantity of ammunition in the entrenchments and pits. In a subsequent telegram Marshal Oyama states that the Russian casualties from the battle at An-shan-chau to the fall of Liau-yang were over 25,000; and an official report from Tokio dated September 10th, gives the total Japanese casualties since August 26th at 17,539. A telegram from St. Petersburg of the same date states that the combined losses of the Russians and Japanese in the fighting between August 26th and September 5th are estimated at between 60,000 and 70,000. On Tuesday General Kuropatkin telegraphed to the Tsar that on Monday he received no reports of any fighting, and there were no signs of activity on the part of the Japanese.

### The Tibet Treaty Signed.

The treaty with Tibet has been signed at Lhasa, the seals affixed being those of Dalai Lama, the Council, the Great Monasteries, and the National Assembly. After the signing of the treaty at the Potala, Colonel Younghusband declared that the British would not interfere with the religious or internal affairs of Tibet. They only desired the establishment of trade relations and the respectful treatment of British representatives and subjects. He hoped that peace would be permanent, but said that any infringement of the treaty would be severely punished. The release of prisoners of war was announced. The arrangements for the return journey of the Mission are now complete, and store depôts having been erected at various points along the route, the only real obstacle to the march is the Tsanpo, which is flowing with tremendous volume. It is expected, however, that its waters will greatly subside in the course of a week or two.

### The New Bishop of Southwell.

The Right Rev. Edwyn Hoskyns, Bishop Suffragan of Burnley, has been appointed Bishop of Southwell in succession to the late Dr. Ridding. The new Bishop, who was born in 1856, and is the son of the Rev. Sir John Leigh Hoskyns, commenced his clerical career as curate of Welwyn, Herts. His first incumbency was that of St. Clement's, North Kensington, and from 1884 to 1895 he was rector of St. Dunstan's, Stepney, where he made himself known as one of the most successful of the East End clergy. In 1895 he accepted the vicarage of Bolton, and four years later he became honorary Canon of Manchester. In 1901 he was advanced to the dignity of Bishop Suffragan and became rector of Burnley at the same time.

### Return of the Antarctic Exploration Ship.

On Saturday the Antarctic ship *Discovery* arrived at Portsmouth, on her return from her voyage of over three years in South Polar regions. There was no formal reception, but Sir Clements Markham, President of the Royal Geographical Society, and a number of dockyard officials went aboard, and gave a warm welcome home to Captain R. F. Scott and his gallant crew. All were deeply bronzed, and were in most cases stouter than when they left England. The ship, like its occupants, appeared to be none the worse for her prolonged stay in the Antarctic. On Sunday morning Captain Scott received a telegram from the King, offering his congratulations on the success and safe return of the exploring party, and expressing his pleasure that the crew had come back in excellent health. It is also intimated by the Admiralty that the King has been graciously pleased to direct that a new medal for service in the Polar regions should be struck and granted to the officers and men of the *Discovery*, in recognition of the successful accomplishment of their enterprise.

### Outrage in Westminster Abbey.

A disgraceful outrage was perpetrated in Westminster Abbey on Sunday morning during the progress of the service. Canon Duckworth had just commenced the office of Holy Communion, when a series of loud explosions were heard. The detonations proceeded from the north transept, close to the door, and resounded loudly throughout the Abbey. The congregation were naturally perturbed, but there was no panic, and only a few persons left their seats. Canon Duckworth requested those who desired to leave to pass along the nave, so as to avoid any confusion, and then proceeded with the office. It was subsequently discovered that two large "crackers" or fireworks, had been placed on the ledge of the monument to the Duke of Newcastle. The fuses, it is surmised, were lighted during the singing of the hymn before the celebration of the Communion, when the striking of a match would not have been noticed. The remains of the fireworks were found in a piece of cardboard, attached to which were a string and a fragment of paper bearing words, including "Vengeance for the death of Kensit," "Lying priesthood," "Enemies of the people." No damage was done to the monument except a few stains caused by the powder.

### A New Version of "Cinderella."

There has just been produced at the Vaudeville Theatre under the title of "The Catch of the Season," the old story of "Cinderella." Of course, it is told in a fresh form, but Angela, the daughter of Sir John Crystal, is treated by her step-mother and sisters in much the same way as the heroine of the popular fairy tale. Lady Caterham is an excellent imitation of the Fairy Godmother, and the Prince is represented by the young Duke of St. Jermyns. There are, of course, departures in detail from the original plot, but only such as are necessary to render it acceptable in the twentieth century, and a most successful result is achieved. "The Catch of the Season" is correctly described as a musical comedy, and the songs and duets are not less attractive than the lively dialogue, Miss Zena Dare delightfully fills the part of the heroine, Mr. Seymour Hicks is the Duke, and Mr. Sam Sothern, the son of the actor who made the character of Lord Dundreary famous, is highly diverting in a similar rôle with the familiar name.

### Notes and Queries.

**FOR REGULATIONS SEE PAGE 143.**

*Dressing.*

(181) Will you kindly tell me if you know of an absorbent dressing for a case of colotomy that is efficacious and less extravagant than gauze tissue or woodwool, etc.?—*E. S.*

Moss pads are the most economical dressings for the purpose you mention. The size of a moss pad is 15 inches by 12½ inches. They can be cut to any smaller size, and should be covered with one layer of plain white gauze.

*Male Nurse.*

(182) I wish to become a male nurse; will you kindly advise me how I can obtain such a post? I should prefer infectious or mental work.—*C. F. W.*

Advertise in any of the principal medical papers, or the *Asylum News*, for an appointment in an asylum for the insane, where the attendants are prepared for the certificate of the Medico-Psychological Association.

*Head and Pelvis.*

(183) Will you kindly tell me where I can obtain, either by purchase or hire, a female pelvis and a fœtal head?—*A Reader.*

Write to the Secretary of the Midwives Institute, 12 Buckingham Street, Strand, W.C.

*Hospital Training.*

(184) Will you kindly tell me if a lady 25 years of age could obtain training in Australia?—*A. C.*

Write to the Hon. Secretary, the Victorian Trained Nurses' Association, St. Ives Private Hospital, Melbourne.

What steps should I take to become probationer in a hospital? My age is 20, and I have a good insight into hospital life.—*A. C.*

You are too young for training in general nursing. See the "Nursing Profession: How and Where to Train" for particulars of children's and special hospitals for which you might be eligible.

Will you kindly tell me the value in poor law of a nurse trained in this hospital? It contains 22 beds and has a large out-patient department, and also maintains a house surgeon.—*K. E.*

With so few beds it can only be a minor training school. Write and ask the Secretary, the Local Government Board, Whitehall, exactly what status the certificate would give to the nurse holding it, and if the qualification would be recognised as sufficient for a superintendent nurse.

Will you kindly tell me how many beds Edmonton Infirmary, London, contains, and say if it is a training school for nurses? Would they, after training, be eligible for post in any general hospital?—*E. H.*

See reply to *K. E.*

*Hospitals.*

(185) Will you kindly tell me which general hospital or hospitals in the British Isles are considered the most up-to-date, and which contain all the latest improvements?—*Isabel.*

The larger hospitals are, as a rule, the most up-to-date.

*Abroad.*

(186) I should be glad to have names and addresses of English nursing homes in Switzerland. Is there not one near Vernay and one at Davos Dorf?—*Beatrice.*

The Davos Invalid Home, Davos Dorf, Switzerland, is the only institution of which we have particulars.

*London County Council Nurses.*

(187) Can you give me any information concerning the nurses who are to be appointed under the London County Council? To whom should one apply?—*Nurse M. B. and Nurse C. J. W.*

Apply to the London County Council, Spring Gardens, S.W.

**Handbooks for Nurses.**

"How to Become a Nurse: The Nursing Profession." 2s. net; 2s. 4d. post free.

"The Nurses' Dictionary." By Honnor Morten. 2s. post free.

"A Handbook for Nurses." By J. K. Watson, M.D. 5s. net; 5s. 4d. post free.

"A Practical Guide to Surgical Bandaging and Dressings." By W. Johnson Smith, F.R.C.S. 2s. post free.

"The Art of Massage." By A. Creighton Hale. 6s. post free.

"Preparation for Operation in Private Houses." By Stanmore Bishop, F.R.C.S. 6d. post free.

The above works are obtainable of all booksellers, or direct from The Scientific Press, Limited, 28 & 29 Southampton Street, Strand, London, W.C.

### For Reading to the Sick.

"NEW HEAVEN AND A NEW EARTH."

Now fain my heart would joyous sing
That lovely summer-time,
When God reneweth everything
In His celestial prime;
When He shall make new heavens and earth,
And all the creatures there
Shall spring from out that second birth,
All glorious, pure, and fair.

The perfect beauty of that sphere
No mortal tongue may speak,
We have no likeness for it here,
Our words are far too weak;
And we must wait till we behold
The hour of judgment true,
That to the soul shall all unfold
What God is, and can do.

*T. Walthers*, 1557.

Heaven is no confined locality, but is wherever God, who is everywhere, can manifest Himself fully to creatures fully capable of Him. And so "the glory which shall be revealed in us" shall be accompanied by a glorious regeneration of our ancient home.

The loveliness of nature, which, even in its temporary obscuration and almost ruin, we have loved, and for which, as the wreck of Paradise, we have blessed our and its Creator—though we have discerned, and that but in transient glimpses, the fringe of His garment, the outer skirts of His magnificent beauty and His almighty power—shall be restored and revealed, and that beyond that primal beauty, even in its first beginnings,

The morning stars sang together,

And all the sons of God shouted for joy.

Such was the faith of the toiling and despised Apostle, whose own "bodily presence was weak and his speech contemptible;" such his hope, as he laboured for the uplifting of the outcast in the cities of the pagan East, or, for two whole years a weary prisoner in the imperial city.

In full and, indeed, in exclusive agreement with the revelation that the scene of man's consummated life in the Resurrection will be the regenerated earth, "a new heaven and a new earth wherein dwelleth righteousness," is the statement that "the holy city, the great city," the "new Jerusalem," the "holy Jerusalem" was seen "coming down from God out of Heaven, descending out of Heaven from God, having the glory of God"; filled, that is, with the Shekinah-brightness of His manifested presence.—*P. G. Medd.*

O Christ, our Refuge and Strength, Thou Hope of human-kind, Whose light shineth from afar upon the dark clouds which hang around us: behold Thy redeemed ones cry unto Thee, Thy banished ones whom Thou hast redeemed with Thine own most precious Blood. Hear us, O God our Saviour, Thou Who art the Hope of all the ends of the earth, and of them that remain in the broad sea. We are tossed about on the wild and stormy waves in the dark night; and Thou, standing on the eternal shore, beholdest our peril: save us for Thy Name's sake. Guide us among the shoals and quicksands which beset all our course, and bring us at length in safety to the Haven where we would be.—*St. Augustine.*

